# Screen-Time Weight-loss Intervention Targeting Children at Home (SWITCH): a randomized controlled trial

**DOI:** 10.1186/s12966-014-0111-2

**Published:** 2014-09-10

**Authors:** Ralph Maddison, Samantha Marsh, Louise Foley, Leonard H Epstein, Timothy Olds, Ofa Dewes, Ihirangi Heke, Karen Carter, Yannan Jiang, Cliona Ni Mhurchu

**Affiliations:** National Institute for Health Innovation, University of Auckland, Private Bag 92019, Auckland, 1142 New Zealand; UKCRC Centre for Diet and Activity Research, University of Cambridge School of Clinical Medicine, Cambridge, England; Departments of Pediatrics, Community Health and Health Behavior and Social and Preventive Medicine, University at Buffalo School of Medicine and Biomedical Sciences, Buffalo, USA; School of Health Sciences, University of South Australia, Adelaide, Australia; Pacific Health, University of Auckland, Auckland, New Zealand; Heke Consulting, Auckland, New Zealand

**Keywords:** Sedentary behaviour, Overweight, Obese, Television, Video games

## Abstract

**Background:**

Screen-based activities, such as watching television (TV), playing video games, and using computers, are common sedentary behaviors among young people and have been linked with increased energy intake and overweight. Previous home-based sedentary behaviour interventions have been limited by focusing primarily on the child, small sample sizes, and short follow-up periods. The SWITCH (Screen-Time Weight-loss Intervention Targeting Children at Home) study aimed to determine the effect of a home-based, family-delivered intervention to reduce screen-based sedentary behaviour on body composition, sedentary behaviour, physical activity, and diet over 24 weeks in overweight and obese children.

**Methods:**

A two-arm, parallel, randomized controlled trial was conducted. Children and their primary caregiver living in Auckland, New Zealand were recruited via schools, community centres, and word of mouth. The intervention, delivered over 20 weeks, consisted of a face-to-face meeting with the parent/caregiver and the child to deliver intervention content, which focused on training and educating them to use a wide range of strategies designed to reduce their child’s screen time. Families were given Time Machine TV monitoring devices to assist with allocating screen time, activity packages to promote alternative activities, online support via a website, and monthly newsletters. Control participants were given the intervention material on completion of follow-up. The primary outcome was change in children’s BMI z-score from baseline to 24 weeks.

**Results:**

Children (n = 251) aged 9–12 years and their primary caregiver were randomized to receive the SWITCH intervention (n = 127) or no intervention (controls; n = 124). There was no significant difference in change of zBMI between the intervention and control groups, although a favorable trend was observed (−0.016; 95% CI: −0.084, 0.051; p = 0.64). There were also no significant differences on secondary outcomes, except for a trend towards increased children’s moderate intensity physical activity in the intervention group (24.3 min/d; 95% CI: −0.94, 49.51; p = 0.06).

**Conclusions:**

A home-based, family-delivered intervention to reduce all leisure-time screen use had no significant effect on screen-time or on BMI at 24 weeks in overweight and obese children aged 9–12 years.

**Trial registration:**

Australian New Zealand Clinical Trials Registry

Website: http://www.anzctr.org.au

Trial registration number: ACTRN12611000164998

## Background

Children and parents alike tout consumption of screen media as central to helping children meet educational, social, and entertainment needs [1–3]. As such, screen-based sedentary behaviours (e.g., watching television [TV], playing video games, and using computers) are common among young people, with 53% of New Zealand children failing to meet guidelines of <2 hours of TV per day [4]. This is of concern given the positive associations between increased levels of screen time, sedentary behaviour, and adverse health outcomes [5].

Sedentary behaviour may be defined as any waking behaviour characterised by low energy expenditure (i.e. ≤1.5 METS) in a sitting or reclined position [6]. Total sedentary time may include screen-based (e.g. watching TV, or using a computer) and non-screen-based pursuits (e.g. reading, doing homework, or motorised transport). However, with respect to health outcomes, not all sedentary behaviours are equal. For example, while reading a book, a traditional or non-targeted sedentary behaviour, has been shown to lower blood pressure [7], screen-based sedentary behaviours have been shown to have adverse effects on overweight and obesity [8–13], metabolic risk [14], attention [15], pro-social behaviour, self-esteem, and academic achievement [16].

A large number of interventions have been conducted to try and address childhood overweight through reductions in sedentary time. While systematic reviews [17–24] of such interventions have tended to report positive, albeit small effect sizes, a number of these reviews have flagged parental involvement and targeting of the home environment as key aspects related to intervention success [21,23].

Active involvement of the parent may be particularly important in ensuring the effectiveness of screen-time interventions. Screen-based activities are highly enjoyable and have an exceptional ability to capture and hold children’s attention. Parents report taking advantage of these unique capabilities by using screens as both an electronic babysitter and an educational tool [2,25]. Furthermore, physical and interpersonal factors within the home environment have been linked to increased screen time in children [26,27]. Together, these factors represent unique barriers, but also unique opportunities for implementing effective and sustainable sedentary screen-time interventions.

To date, a number of home-based sedentary behaviour interventions involving a parental component have been conducted in children and adolescent populations [28–35]. Most of these interventions have focused on the child, with parents primarily concerned with overseeing implementation of various devices, such as TV electronic time monitors [28] and active video games [32,33]. Furthermore, study sample sizes have tended to be small and follow-up too short to gauge the longer term success of such interventions and samples have been relatively homogeneous in terms of ethnicity [28]. In an attempt to address these shortcomings, we conducted a large, 6-month randomised controlled trial to investigate the effectiveness of a home-based, family-delivered intervention aimed at decreasing screen-based sedentary behaviour and improving body composition in overweight and obese children. The intervention focused on training and educating primary caregivers to use a wide range of strategies designed to reduce their child’s screen time.

## Methods

The Screen-Time Weight-loss Intervention Targeting Children at Home (SWITCH) study was a parallel, two-arm, randomized controlled trial aimed at preventing excessive weight gain through reductions in screen-based sedentary activities. The trial was undertaken in New Zealand between 2010 and 2012 [36]. Participants were eligible if they were children aged 9–12 years, lived in the greater Auckland metropolitan area, used electronic media (television, video games, computer) for at least 15 hours per week, were overweight or obese (as per Cole International cut-points) [37], and could speak and understand English. Given the high prevalence of obesity among Māori (Indigenous [19%]) and Pacific children (26%) [38], we aimed to recruit equal numbers of Māori, Pacific and non-Māori/non-Pacific children. Participants were asked to self-select the single ethnic group they most identified with. In New Zealand the majority of Māori and Pacific live in the greater Auckland region, integrated within existing communities. While there are greater health disparities for Maori and Pacific compared to non-Māori/non-Pacific, physical activity levels and leisure time pursuits are similar for these populations. One child per household was eligible to participate in the study. Children were excluded if they had any medical condition that precluded them from participating in regular physical activity or if they lived in more than one household and spent equal time at both households. Each participating child was also required to have one primary caregiver take part in the study that was aged 18 years or older, and could speak and understand English. Participants were recruited via schools, community centres, churches, primary healthcare organisations and word of mouth. Ethical approval for the trial was obtained from the Lower South Regional Committee (LRS/10/09/039). Written informed consent was provided by caregivers and written assent was provided by children.

### Randomisation and blinding

Eligible participants were randomised at a 1:1 ratio to the intervention or control groups via centralised computer randomisation, using stratified blocked randomisation (with variable block sizes) to maintain balance across important prognostic factors. Two stratification factors were considered: sex (male and female) and ethnicity (Māori, Pacific, and non-Māori/non-Pacific). Allocation concealment was maintained up to the point of randomisation. Blinding of participants and research assistants was not possible due to the nature of the intervention.

### Procedure

Assessments were undertaken at the participant’s home at baseline and 24 weeks post-randomisation. At the baseline visit, written informed consent was obtained and then physical measurements of the child’s height, weight, waist circumference, and body composition were recorded. Maturation status was not assessed in the present trial due to reasons of logistics and issues of cultural sensitivity. Self-reported measures of sedentary behaviour, physical activity, diet, and enjoyment of sedentary behaviour and physical activity were also assessed. Height and weight of the primary care giver were measured and then a 7-day physical activity questionnaire was administered. All measures were repeated at the 24-week visit. Participants in both groups also received a phone call at 12 weeks to monitor any adverse events.

### Intervention

The intervention focused on reducing leisure time screen-based sedentary behaviour in children and was delivered over 20 weeks. It was based on a previous trial conducted in the United States (U.S.) by Epstein et al. [28], which used TV restriction monitors, education, multiple home visits and incentives to reduce young children’s (aged 7–9 years) TV viewing and computer use. This study was associated with sustained changes over the two year period in screen-based sedentary behavior. The U.S. intervention content was adapted for use in New Zealand and included a broader focus on all screen-based activities in the home (TV, video and to start after computer use). Adaptation also included the removal of financial incentives from the intervention, as this was not considered a scalable approach in New Zealand, modification of content to ensure its acceptability for Māori (indigenous) and Pacific families, and the inclusion of a participant website to facilitate information sharing.

Intervention content was grounded in Social Cognitive Theory (SCT) [39] and behavioural economics theory (BET) [40] and details are provided below. SCT refers to the triadic, reciprocal relationship between the personal, environmental, behavioral factors and the environment [39]. SCT explains how people acquire and maintain certain behavioral patterns, while also providing the basis for intervention strategies [39]. In the present intervention we focused on implementing changes to the home and family environment, as well as providing suggestions for parents to make personal and behavioral changes (outlined below). According to BET, choices to engage in a physical activity involves choosing this over a competing sedentary behaviour [41], for example choosing to walk to school rather than drive with a parent. This choice is dependent on two factors, the relative ease of access to the competing activities and their reinforcing (enjoyment) value. A key tenet of BET is that modifying one behaviour will result in subsequent changes to another related behaviour [41]. BET posits that physical activity and sedentary behaviour are related as behavioural substitutes. Therefore, decreasing time spent being sedentary may lead to substitution with physical activity as this time is reallocated [41]. However, the strength of the inverse relationship between physical activity and sedentary behaviour (“cross-elasticity of time use”) depends on the specific activities and time blocks in question. Furthermore, complete substitution of sedentary time with moderate to vigorous physical activity will likely not occur; one sedentary behaviour (e.g., TV watching) could be substituted with another (e.g., reading), or with light intensity physical activity. Intervention studies conducted in children indicate that reducing a targeted sedentary behaviour (screen-time) was associated with significant increases in physical activity, though non-targeted sedentary behaviours also increased [42–45]. A key focus was to train the primary caregivers to initiate change in the home environment to facilitate behaviour change of the child and to implement the behaviour change strategies. Three elements were offered to families: (1) provision of behaviour change strategies, (2) assistance to budget media time, and (3) an activity pack for children.

### Provision of behaviour change strategies

Primary care givers were offered education and support to implement strategies in the home environment to reduce media use. While only one child per household was assessed as part of the SWITCH trial, the intervention was focused on the entire home environment (consistent with SCT). During an initial face-to-face meeting, trained research assistants provided culturally relevant education to the primary caregiver. Role modelling and observational learning are key tenets of SCT, and reinforcing value is a key determinant of behaviour choice according to BET. As such, the intervention aimed to modify both of these determinants to reduce screen time. During the face-to-face meeting, caregivers were provided an overview of the intervention and encouraged to include praise, positive reinforcement, environmental control budgeting and self-monitoring, positive role modelling, and offer alternative activities to screen-based media. Families also received brief monthly newsletters outlining additional strategies for reducing screen-based sedentary activities, which included a description and rationale for using the respective strategies, and practical advice about implementing these. Different versions of each newsletter were available for Māori, Pacific and non-Māori/non-Pacific families. While the content of the newsletters was the same for each group, the visual presentation and language differed. A secure website was also provided to support the intervention content, and included the monthly newsletters in electronic format, additional tips and information for reducing screen-based activity and alternative options (such as playing board games, doing homework, arts and crafts, using one of the items from the SWITCH activity pack [see below], playing outside and being physically active), and links to community-based activity programs [34].

### Budgeting media use

Based on previous pilot work [34], families in the intervention group each received two Time Machine (Family Safe Media, Park City, US) TV monitoring devices to help budget their media use. The Time Machine was connected to a TV, or other media device (e.g., DVD player, video game console), but it was not possible to connect the device to a computer. The Time Machine cables were secured and locked to prevent tampering, and parents were given keys to access the cables if need be. Each Time Machine came with 30 tokens, with each token allowing 30 minutes of viewing time; however caregivers were able to allocate these as they chose.

### Activity pack

Children in the intervention group were given an activity pack containing suggestions and options for non-screen based activities and included include coloring pencils, a length of rope, playing cards, a tennis ball for playing handball or similar games, and activity cards, which described simple games or activities to play. Further adaptation of the intervention also included offering children Māori-specific board games and instructions for traditional Māori games.

### Fidelity of delivery of the intervention

Monitoring of the intervention delivery by community workers was undertaken. A member of the research team attended a face-to-visit, and observed each of the community workers delivering the intervention content and recorded the absence or presence of techniques discussed using a standard format. At the end of the assessment, the researcher provided feedback to the community worker to ensure all components of the intervention were delivered.

### Control

The control group was asked to continue with their usual behaviour and had access to a generic SWITCH public website. The primary caregiver in both groups was contacted at 12 weeks to confirm contact information and record any serious adverse events. At the end of the study follow-up, the control group were offered the intervention components.

### Outcomes

All outcomes were assessed at baseline and 24 weeks.

### Primary outcome

The primary outcome was change in children’s BMI z-score (standardized by age and sex using 2007 WHO growth reference), from baseline to 24 weeks.

### Secondary outcomes

Child: BMI (kg/m^2^), body weight (kg), waist circumference (cm), percentage body fat (%), self-reported measures of daily physical activity (PA; including minutes of total PA, light intensity PA [LPA] and moderate-to-vigorous PA [MVPA]), total sedentary time (minutes), sleep, dietary intake (daily energy intake [KJ], energy consumed from snacks, and frequency of soft drink consumption), and perceived enjoyment of physical activity and sedentary behaviour.

Primary caregiver: BMI and self-reported physical activity (daily minutes of total PA, LPA, and MVPA).

### Measures

#### Anthropometrics

Anthropometric measurements were conducted according to standard practices [46]. BMI was calculated from height and weight data (kg/m^2^) and converted to a standardised z-score using age- and sex-specific 2007 WHO growth reference for 5–19 years (www.who.int/growthref). Body composition was assessed via bioelectrical impedance using the ImpediMed DF50 Bioimpedence Monitor (Queensland, Australia). Fat free mass (FFM), fat mass (FM), and percentage body fat were calculated for all participants using New Zealand-specific equations [47].

#### Physical activity and sedentary behaviour

Children’s physical activity and sedentary behaviour were measured using the Multimedia Activity Recall for Children and Adolescents (MARCA) [48], a computerised, self-report, 24-hour use-of-time recall that uses a segmented day format with self-determined anchor points. The MARCA has same-day test-retest reliability of r =0.84-0.92 for major outcome variables, and validity with reference to accelerometry of r = 0.45 for physical activity level (PAL) [48]. Children were asked to recall their activities for the two previous days (48 hours). When the child reported taking part in two activities simultaneously, the following hierarchy was used for analysis purposes: physical activity, screen time, active transport, passive transport, and “other” activities. For example, if a child reported watching a DVD at the same time as being driven somewhere, the activity would count as screen time. Time spent in each activity was summed to determine how much time each participant spent in total PA, LPA (≥1.5 METs and <3 METs), MPA (≥3 METs and <6 METs), vigorous physical activity (VPA; ≥6 METs), locomotion, total sedentary time (non-sleep activities <3 METs), screen-based sedentary time, non-screen sedentary time, and sleep.

Primary caregiver PA was measured using the International Physical Activity Questionnaire long form (IPAQ-LF) [49], which assesses domain-specific physical activity, walking and sitting. MET-minutes per week were calculated as duration × frequency per week × MET value, which was summed across activity domains to produce an estimate of total PA from all reported activities.

#### Dietary intake

A semi-quantitative food frequency questionnaire (FFQ) was used to record information on dietary intake. The FFQ was developed for the New Zealand Children’s Nutrition Survey [50] and has similar repeatability to child and adolescent FFQs used in other countries [51]. It comprised 104 commonly consumed food items. Children were asked how often, and how much they’d eaten of each of the foods over the previous four weeks. Through use of standardised portion sizes, total daily energy intake was estimated, as well as energy intake from snack foods. Consumption of specific food items (e.g., snack foods, and sugar-sweetened beverages) was also recorded.

#### Enjoyment of physical activity and sedentary behaviour

Psychological variables were measured to determine their potential mediating effect. Perceived enjoyment of physical activity was assessed using the 14-item Physical Activity Enjoyment Scale [52], adapted for use in adolescents in 2001 [53]. Perceived enjoyment of sedentary behaviour was assessed using a scale adapted from Salmon et al. [54].

#### Process evaluation

Primary caregivers involved in the intervention completed an exit survey to determine their perceptions of the intervention and their use of the intervention components.

### Sample size

An *a priori* sample size estimate indicated that 270 children (135 per arm) would provide at least 90% power at the 5% level of significance (two-sided) to detect a 0.2 unit difference in change of zBMI from baseline to 24 weeks between the intervention and control groups, assuming a standard deviation of 0.5. The sample also would provide 80% power to detect a 0.3 unit difference in change of zBMI between ethnic groups (Māori, Pacific, and non-Māori/non-Pacific).

### Statistical analysis

A formal statistical analysis plan (SAP) was approved by the Trial Steering Committee before datalock. Statistical analyses were performed using SAS version 9.3 (SAS Institute Inc. Cary NC) and R version 2.15 (R Foundations for Statistical Computing). All statistical tests were two-tailed and a 5% significance level maintained throughout the analyses. Treatment evaluations were performed on the principle of intention to treat (ITT), using data collected from all randomised participants. A multiple imputations method was applied to the missing data (if any) for the primary outcome. No imputation was undertaken for secondary outcomes. Analysis of covariance (ANCOVA) regression models were used to evaluate the main treatment effects on primary and secondary outcomes at 24 weeks, adjusting for baseline outcome value, age, sex, and ethnicity.

## Results

Four hundred and four children registered to the SWITCH trial, with 251 (62%) eligible to participate. Children were randomly assigned to the intervention (n = 127) and control (n = 124) groups, with 121 (95%) and 117 (94%) completing 24 weeks’ follow up. The majority of children were male (57%) and of Pacific origin (n = 133, 53%), with a mean age of 11 years. Treatment groups were well balanced in terms of baseline characteristics (Table 1).

**Table 1:** **Study participants**

	**Intervention Mean (SD)**		**Control Mean (SD)**		**Treatment difference at 24 weeks (Intervention - Control)**	
	Baseline (n = 127)	24 weeks (n = 117)	Baseline (n = 124)	24 weeks (n = 113)	Estimate (95% confidence interval)	P-value
**Demographics**						
Age (years)	11.2		11.3			
Gender:						
Male	72 (57%)		70 (56%)			
Female	55 (43%)		54 (44%)			
Ethnicity:						
Māori	16 (13%)		13 (11%)			
Pacific	67 (53%)		66 (53%)			
NZ/European	44 (34%)		44 (35%)			
Refused to answer	0		1 (1%)			
**Total household income (before tax)**						
Under $20,000	14 (11%)		22 (18%)			
$20,001-$30,000	14 (11%)		21 (17%)			
$30,001-$40,000	19 (15%)		17 (14%)			
$40,001-$50,000	18 (14%)		14 (11%)			
$50,001-$60,000	5 (4%)		9 (7%)			
$60,001-$70,000	11 (9%)		4 (3%)			
$70,001-$80,000	9 (7%)		7 (6%)			
$80,001-$90,000	6 (5%)		6 (5%)			
Over $90,000	19 (15%)		12 (10%)			
Don’t know	9 (7%)		12 (10%)			
Refused to answer	3 (3%)		0 (0%)			
**Anthropometrics**						
zBMI score	2.57 (0.8)	2.58 (0.86)	2.52 (0.93)	2.56 (0.94)	−0.01 (−0.08, 0.05)	0.67
BMI (kg/m^2)^	26.51 (4.50)	26.63 (4.69)	26.62 (5.30)	26.75 (5.19)	−0.01 (−0.36, 0.35)	0.97
Height (m)	1.53 (0.10)	1.57 (0.10)	1.54 (0.10)	1.57 (0.10)		
Weight (kg)	63.21 (15.92)	66.01 (16.64)	63.98 (18.50)	66.57 (18.36)		
Waist circumference (cm)	87.45 (14.39)	89.06 (13.30)	87.89 (14.79)	89.12 (13.75)	−0.09 (−1.95, 1.77)	0.93
FFM (kg)	42.01 (9.23)	44.08 (8.94)	42.48 (10.11)	44.54 (0.47)	0.24 (−0.63, 1.11)	0.59
FM (kg)	21.20 (8.01)	21.85 (9.31)	21.05 (10.11)	21.24 (9.21)	0.08 (−0.97, 1.14)	0.88
Percentage body fat (%)	32.78 (6.06)	32.14 (6.76)	31.93 (9.92)	31.41 (6.93)	−0.05 (−1.38, 1.27)	0.94
**Sedentary behaviours**	Baseline (n = 127)	24 weeks (n = 110)	Baseline (n = 124)	24 weeks (n = 105)		
Total sedentary time (min/day)	550 (136)	520 (130)	561 (136)	542 (140)	−20 (−56, 17)	0.29
Screen-based sedentary time (min/day)	250 (162)	120 (147)	244 (145)	235 (156)	−33 (−73, 7)	0.11
Non screen-based sedentary time (min/day)	299 (137)	321 (133)	317 (137)	307 (150)	13 (−26, 51)	0.51
Enjoyment of sedentary behaviour	3.69 (0.62)	3.61 (0.54)	3.80 (0.55)	3.78 (0.64)	−0.12 (−0.26, 0.02)	0.09
**Physical activities**	Baseline (n = 127)	24 weeks (n = 110)	Baseline (n = 124)	24 weeks (n = 105)		
PA level (MET/day)	1.58 (0.26)	1.59 (0.25)	1.64 (0.34)	1.60 (0.32)	0.01 (−0.07, 0.09)	0.82
LPA (min/day)	109 (59)	129 (81)	114 (72)	138 (89)	−9 (−33, 14)	0.43
MPA (min/day)	113 (96)	113 (94)	111 (104)	89 (94)	24 (−1, 50)	0.06
VPA (min/day)	31(45)	32 (48)	44 (62)	37 (52)	−1 (−15, 12)	0.84
MVPA (min/day)	144 (100)	145 (100)	155 (110)	127 (109)	22 (−6, 51)	0.13
Locomotion	164 (103)	174 (101)	174 (119)	165 (108)	9 (−20, 38)	0.54
Sleep (min/day)	640 (85)	645 (95)	619 (85)	633 (99)	8 (−19, 34)	0.57
Enjoyment of physical activity	4.13 (0.52)	4.18 (0.57)	4.21 (0.58)	4.24 (0.59)	0 (−0.1, 0.1)	0.82

LPA: light physical activity; MVPA: moderate-to-vigorous intensity physical activity; SD: standard deviation; VPA: vigorous physical activity. Linear regression models for intervention difference adjusting for: baseline outcome value, age (in years), sex, most identified ethnicity.

Household income is in NZ$: As of June 2014, $NZ1 = $US0.85.

### Primary outcome

At 24 weeks, the mean changes from baseline in zBMI were 0.03 and 0.05 in the intervention and control groups, respectively, with a between-group difference of −0.01 (95% C.I. -0.08 to 0.05), which was not statistically significant (p = 0.64). The results were similar using multiple imputations on the small proportion of missing data, with a group difference of −0.01 (95% C.I. -0.08 to 0.05; p = 0.67).

### Secondary outcomes

#### Anthropometrics

There were no significant changes in BMI, waist circumference, fat free mass, fat mass, percentage body fat, or parent/caregiver BMI between intervention and control groups at 24 weeks (Table 2).

**Table 2:** **Treatment difference at for primary caregivers 24 weeks (intervention - control)**

**Outcome**	**Estimate (95% confidence interval)**	**P-value**
BMI (kg/m^2^)	−0.36 (−0.96, 0.24)	0.24
Total physical activity (MET min/week)	−172.72 (−1793.72, 1448.48)	0.83
Occupational physical activity (MET min/week)	−222.50 (−1489.50, 1044.49)	0.73
Active transport (MET min/week)	−226.46 (−610.58, 157.66)	0.25
Domestic and gardening (MET min/week)	413.04 (−294.15, 1120.23)	0.25
Leisure time (MET min/week)	−41.47 (−502.76, 419.82)	0.86
Walking (MET min/week)	−205.23 (−783.37, 372.90)	0.48
MPA (MET min/week)	345.86 (−506.60, 1198.33)	0.42
VPA (MET min/week)	−185.01 (−1117.05, 747.04)	0.67

#### Physical activity, sedentary behaviour and sleep

At the end of the 24-week intervention there were no significant differences in measures of physical activity, sedentary behaviour, or sleep between intervention and control groups. However, there was a trend for increased MPA from baseline in the intervention group at 24 weeks (+24.3; 95% CI: −0.94, 49.51; p = 0.06). At 24 weeks, children in both the intervention and control groups spent the majority of their time engaged in sedentary behaviours (approximately 8.9 hours/day). Although not statistically significant, both groups reported decreased sedentary time at 24 weeks (Table 1).

Given the focus of the intervention was to modify leisure time screen activities, a sensitivity analysis was conducted to determine the effect of the intervention on after school sedentary (screen and non-screen) time and physical activity levels. Same regression analyses were conducted examining use-of-time data for weekdays between 15:00 and 18:00 hours as well as weekends; however no significant differences were observed.

Children’s enjoyment of physical activities and sedentary behaviours did not differ significantly after the intervention.

### Dietary intake

After week 24, there were no differences in self-reported dietary intake between the intervention and control groups.

### Primary caregiver

There were no significant differences between intervention and control groups in caregiver BMI, physical activity, or sedentary behaviour levels after the 24-week intervention (Table 2).

### Process evaluation

Almost half (46%) of participants reported never using the Time Machine to budget their child’s television or computer use; however 57% reported using any of the strategies discussed in the monthly newsletters. In the previous week, 43% of the caregivers reported using any of the strategies to modify screen use sometimes to often.

## Discussion

A 24-week, home-based, family-delivered intervention had no significant effect on zBMI in 9- to 12-year-old children in New Zealand. Further, the intervention was not associated with significant changes in physical activity, sedentary behaviour, sleep, dietary intake, or preferences of physical activity and sedentary behaviours. There was also a null effect of the intervention on primary caregivers’ BMI, physical activity, sedentary behaviour, and activity preferences. Despite these null findings, there were consistent trend effects for most of the self-reported measures of interest, including decreases in total sedentary time, screen-based sedentary time, and total energy intake, as well as increased MVPA. Taken together, this suggests the intervention was not intensive enough to manipulate these behaviours and positively impact on body mass.

### Strengths and limitations

Strengths of the study included the randomized design, collection of physical activity data from the child and their primary caregiver, use of measurement tools (MARCA and FFQ) that have previously been validated in similar populations, adequate sample size to detect change in BMI, and a relatively long follow-up. Further, our intervention focussed primarily on reducing sedentary screen time, rather than incorporating other health messages, and utilised a TV locking device in addition to several behaviour modification strategies, two strategies that have recently been identified as important intervention features associated with greater reductions in sedentary screen behaviours in children [22]. Finally, compared to similar trials [28] our sample was more heterogeneous in terms of ethnicity, and included substantial numbers of Pacific and indigenous Māori children, who experience the greatest burden of obesity [4].

A limitation of this trial was the self-reported measures of sedentary time and physical activity. Previous studies have used more objective measures, including TV monitors to capture screen time [28]; however, for logistic reasons this was not possible in our trial. In the present study, while we used a reliable and valid use-of-time recall tool [48], its sensitivity to assess change is less clear. Second, the intervention did not have the intended effect on the targeted behaviours (sedentary time and physical activity), hence the lack of effect on body mass is unsurprising. This lack of effect may be explained by the bias associated with self-report measures, the lack of intensity associated with the intervention, and/or poor compliance. The intervention was delivered in a one-off meeting with the primary caregiver, who was only contacted again after 12 weeks to confirm contact details and monitor adverse events. This may have been insufficient contact time to elicit change in the child. Finally, there were also some technical issues with the Time Machine not working with satellite or pay TV or on household computers, which may have limited the capability of the device to help with family budgeting of screen use.

### In context with previous work

Although this present study was modeled after previous work by Epstein et al. [28] our findings differed considerably. Disparity in our findings may be explained by the following. First, our older sample of children (9–12 years) may be less amenable to parental influence compared to the younger sample (4–7 years) in Epstein’s trial. According to a recent systematic review of family-focused interventions aimed at reducing sedentary behaviours [55], interventions targeting younger children may be more effective at decreasing sedentary time than those targeting older children. Second, we used a different time monitor (Time Machine versus TV Allowance) to budget screen time due to logistic issues associated with voltage differences between the two countries. Although our pilot trial suggested that participants would engage with such technology to modify screen time [34], data from the exit interviews suggested this was not the case. Third, we did not use financial incentives to change behavior as it was not considered to be a scalable delivery option as an ongoing program, or at a population level. While using incentives to affect behavior change may work in the short term, apart from the sustained effects (2-years) observed by Epstein et al. [28] there is little evidence to suggest that incentives promote long-term changes in behavior [56]. From a behavioral economics perspective, the removal of a financial incentive from the intervention may be particularly relevant when explaining differences in outcomes between our study and that conducted by Epstein et al. [28]. Research has shown that children use screen-based media because it is highly valued and rewarding [57], thus trying to substitute this with non-financial rewards, such as playing board games, playing with siblings, or increasing physical activity may be less valued and therefore less successful. In sum, while the content of this intervention was modified from the US trial [28], failure to adhere to the fidelity of the initial program may have accounted for the failure to replicate findings.

Consistent with Epstein et al. [28] our intervention was developed to target aspects of SCT and BET. For example, the intervention encouraged parents/caregiver to act as role models for decreasing screen time. However this may have proven difficult if parents also highly valued screen time such as watching TV. Further, during the face-to-face meeting and via the newsletters, caregivers were encouraged to include praise, positive reinforcement, environmental control budgeting and self-monitoring, and offer alternative activities to screen-based media. While these are key constructs of BET, we are unclear to what extent parents/caregiver implemented these techniques. Exit surveys were conducted, but more intensive monitoring of their implementation was not undertaken for two reasons. First, SWITCH was a pragmatic trial, in which the study was designed to maximize the ecological validity of the findings [58] and more intensive monitoring may have been an intervention in itself. Second, resources required for such intensive monitoring were outside the scope of this trial. We did however undertake monitoring visits to determine the fidelity of delivery of the intervention by the community workers.

Although a number of home-based interventions have been shown to significantly reduce screen time in children, these studies tended to have small sample sizes and longer term effectiveness has not been assessed (except for Epstein et al. [28]). Compared to many previous studies, although SWITCH failed to demonstrate a significant effect on reducing screen time, its follow-up was longer and the sample size substantially larger. In fact, similar null effects on screen time have also been demonstrated in other home-based interventions that have utilised larger sample sizes and longer durations of follow-up [30,32].

SWITCH is one of the few trials to examine the effects of an intervention on both child and caregiver outcomes. Increasingly, there is evidence to support the importance of targeting caregivers in child obesity interventions, as caregiver behaviors affect the child’s behaviours either directly, through sharing behaviours (e.g. watching TV together), or indirectly, through the shared environment [59]. Given the importance of parents/caregivers in influencing the home environment (e.g., setting rules/guidance for household screen-time limits, influencing the physical environment, and purchasing/availability of household foods), it is not surprising that children’s behaviours did not change when there was no observed change in their caregiver’s behaviours or BMI.

### Future directions and implications

There is growing evidence to support active targeting of caregivers in sedentary behaviour interventions; however, level of caregiver intensity appears to be an important determinant of intervention success [55]. As such, despite it being a negative trial, SWITCH may be viewed as a lesson in intervention design. Future studies may need to increase the contact time with caregivers to assist them to elicit change at the family level. Furthermore, exact replication of the Epstein et al. [28] protocol may yield positive results.

While parents acknowledge that children spend a lot of time interacting with screens, they may also avoid limiting screen time for fear of negatively affecting their child’s development. Research shows that parents often cite TV watching and computer use as important educational tools [2]. Further, caregivers may underestimate the severity of children’s media exposure, as high levels of sedentary screen behaviour may now be normalized in society; they may also underestimate the impact of these behaviours on health outcomes. Future research should therefore assess caregiver perceptions regarding screen time and the effects of screen time on health, in order to inform future interventions.

It has been suggested that decreasing sedentary screen time may be easier than increasing physical activity [16]; however, the limited ability of interventions to effectively decrease sedentary screen time is becoming more apparent. Screen-based behaviours are highly rewarding. Recent advances in the miniaturisation and portability of screens, the availability of content that specifically targets young people, as well as the ubiquitous nature of screens (iPads, iPods etc.) mean that opportunities for screen-based exposures have increased exponentially [60]. As such, restricting or budgeting access at the household level is increasingly difficult, as observed in the present study.

## Conclusions

A home-based, family-delivered intervention to reduce all leisure-time screen use had no significant effect on screen-time or on BMI in overweight and obese children aged 9–12 years.

## Abbreviations

SWITCH: Screen-Time Weight-loss Intervention Targeting Children at Home
TV: Television
BMI: Body mass index
METS: Metabolic equivalents
IPAQ-LF: International Physical Activity Questionnaire Long Form
FFQ: Food Frequency Questionnaire
FFM: Fat free mass
FM: Fat mass
MARCA: Multimedia Activity Recall for Children and Adolescents
SCT: Social Cognitive Theory
BET: Behavioural Economics Theory
PAL: Physical activity level
LPA: Light physical activity
MVPA: Moderate-to-vigorous intensity physical activity
VPA: Vigorous physical
